# An Unusual Presentation of Dermatofibroma with Ulcer: A Case Report

**DOI:** 10.3390/dermatopathology12030028

**Published:** 2025-09-04

**Authors:** Lamia Alakrash, Renad AlKanaan, Rema Aldihan, Alanoud Alsuhibani, Salman Almalki

**Affiliations:** 1 Department of Dermatology, King Fahad Medical City, Riyadh 11525, Saudi Arabia; 2 College of Medicine, King Saud University, Riyadh 11461, Saudi Arabia; 3 Department of Dermatology, King Abdulaziz Medical City, National Guard Health Affairs, Riyadh 11426, Saudi Arabia; 4 Department of Pathology, King Fahad Medical City, Riyadh 11525, Saudi Arabia

**Keywords:** dermatofibroma, ulcer, ulcerated lesion, immunocompromised, histopathology

## Abstract

Dermatofibroma is a common mesenchymal skin lesion that typically presents as a firm, slow-growing nodule. Generally, such lesions are asymptomatic; however, they can also cause discomfort in some cases. Ulceration is an uncommon feature of dermatofibroma, and diagnosis in such cases is often difficult. We report a case of a 67-year-old female with multiple comorbidities, including pancreatic cancer undergoing neoadjuvant chemotherapy, who was admitted for acute pulmonary embolism. The patient presented with an incidental medial thigh lesion. The lesion was asymptomatic, ulcerated, and oozing pus one month before presentation. Clinical examination revealed a 3 × 2 cm deep ulcer with a punched-out edge, a dry yellow-white base, and a firm violaceous border. Histopathology confirmed dermatofibroma with epidermal hyperplasia, dermal spindle cell proliferation, histiocytes, and collagen trapping. Immunohistochemistry was positive for CD68, CD10, and Factor XIII. Due to the deteriorating condition of the patient, no intervention was provided to her, and she succumbed to her primary illness. This case is unique due to its atypical ulcerative presentation in a patient with complex systemic illness and emphasizes distinguishing between benign lesions and malignant mimics, especially in cases which have ambiguous clinical presentation.

## 1. Introduction

Dermatofibroma, also known as benign fibrous histiocytoma, is a common benign skin tumor characterized by slow-growing, firm, well-circumscribed nodules. These lesions are often asymptomatic but may occasionally present with pruritus or tenderness [1,2,3]. Dermatofibromas predominantly affect adult women and are frequently located on the lower extremities [4]. Dermatofibromas account for approximately 3% of all excised cutaneous lesions [2]. However, this figure may underestimate the true prevalence, as many lesions remain clinically silent and are not subjected to histopathological examination.

The etiology of dermatofibroma is not fully understood. Local trauma, such as insect bites or surgical scars, is a proposed trigger [5], though reported in only a minority of cases [6]. Immunologic responses, particularly in immunocompromised patients, have also been suggested [7]. Dermatofibromas are typically less than 1 cm but can exceed 3 cm, with rare giant variants over 5 cm. They often exhibit a “dimple sign” upon lateral compression due to attachment to underlying tissue [8]. While they can occur anywhere, the extremities are the most common site, with or without skin discoloration [9]. Recognized variants include cellular, aneurysmal, atypical, and perforating dermatofibromas, each with distinct histological features [9]. Ulceration, as seen in our case, is a rare clinical manifestation rather than a distinct histological subtype [9,10].

Diagnosis typically depends on clinical evaluation and history. Histologically, dermatofibromas are composed of intersecting fascicles of spindle-shaped fibroblasts and histiocytes arranged in a storiform pattern embedded within a collagen-rich stroma [11]. These may be accompanied by overlying epidermal hyperplasia, acanthosis, or hyperkeratosis [12]. In deeper variants, extension into the superficial subcutis is more common [2].

In ulcerated dermatofibromas, histopathological examination is even more critical. Ulceration leads to the disruption of the epidermis and superficial dermis, often accompanied by a reactive inflammatory infiltrate composed of lymphocytes, plasma cells, and neutrophils [13]. Thick sclerotic bundles of collagen fibers interwoven with spindle cells are typical, with peripheral collagen trapping considered a supportive diagnostic feature [14]. Ulceration may also cause necrosis or hemorrhage, complicating interpretation and requiring careful tissue sampling [1].

A panel of immunohistochemical stains is often necessary to rule out malignant mimics. Dermatofibromas usually express Factor XIIIa and are negative for CD34 [15], which helps differentiate them from dermatofibrosarcoma protuberans (CD34-positive and Factor XIIIa-negative) [10]. The gradual histological transition from lesional to normal dermis and the infiltration of spindle cells into surrounding collagen are also distinguishing features [16,17].

This report presents a rare, ulcerated dermatofibroma in an immunocompromised patient, mimicking more aggressive neoplasms [18]. By integrating clinical, histopathological, and immunohistochemical findings, we aim to highlight diagnostic challenges and enhance awareness of this uncommon presentation.

## 2. Case Presentation

A 67-year-old female with hypertension, diabetes mellitus, hypothyroidism, and pancreatic cancer on neoadjuvant chemotherapy was admitted for acute pulmonary embolism. During hospitalization, a dermatology consultation was requested for an incidental lesion on the medial thigh. The patient reported a small, asymptomatic nodule appearing three months prior, which enlarged, ulcerated, and began oozing pus one month before evaluation.

**Figure 1:**
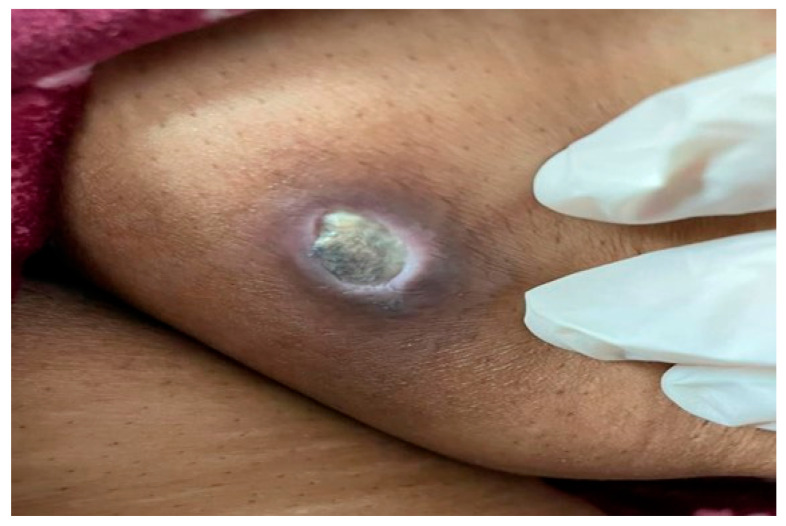
Clinical image showing a non-tender, oval-shaped deep ulcer measuring 3 × 2 cm on the medial aspect of the thigh, characterized by a punched-out edge, yellowish-white dry base, and a firm violaceous border.

Clinical examination revealed a non-tender, oval-shaped, 3 × 2 cm deep ulcer with a punched-out edge, yellow-white dry base, and firm violaceous border (Figure 1). No lymphadenopathy was noted. Differential diagnoses included blastomycosis, ecthyma gangrenosum, and cutaneous metastasis from pancreatic cancer [11].

**Figure 2:**
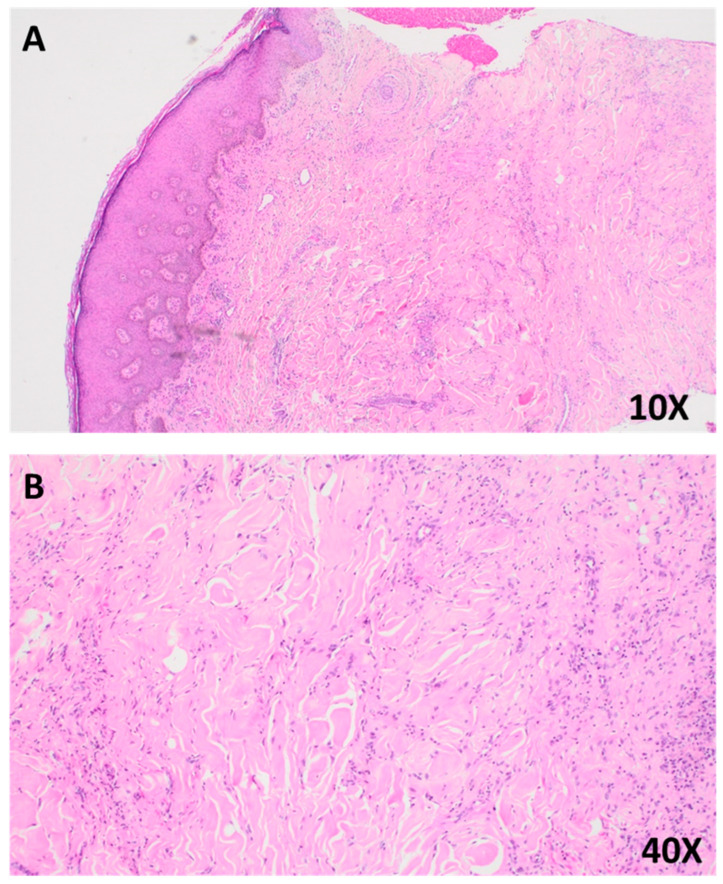
(**A**) Photomicrograph of a hematoxylin and eosin (H&E)-stained section at 10× magnification, showing epidermal hyperplasia with basal layer hyperpigmentation and prominent collagen trapping within the dermis. (**B**) Higher magnification view (40×), highlighting interlacing bundles of spindle-shaped fibroblasts and histiocytes embedded within a collagen-rich stroma, consistent with features of dermatofibroma.

A 4 mm punch biopsy was chosen due to the patient’s frail condition and the need for a minimally invasive procedure to balance diagnostic yield with procedural risk. The histopathology of hematoxylin and eosin (H&E)-stained sections showed epidermal hyperplasia with focal basal layer hyperpigmentation, a non-encapsulated proliferation of spindle-shaped fibroblasts and histiocytes in a storiform pattern, and prominent collagen trapping with sclerotic bundles (Figure 2A,B). These findings confirmed dermatofibroma [19].

**Figure 3:**
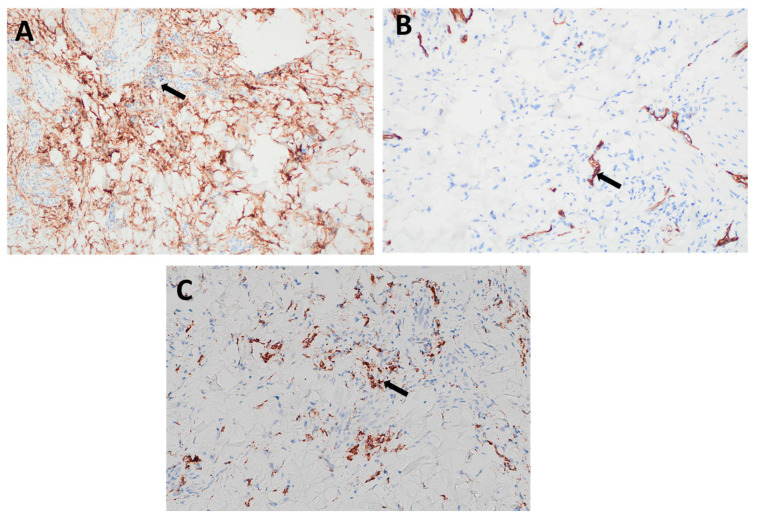
Immunohistochemical staining of the lesion: (**A**) Strong cytoplasmic positivity for CD10 in dermal spindle cells (arrow), supporting a diagnosis of dermatofibroma. (**B**) Negative staining for CD34 in the lesional spindle cells (arrow), helping to differentiate the lesion from dermatofibrosarcoma protuberans, which typically shows strong CD34 positivity. (**C**) Positive staining for CD68 (arrow), indicating the presence of histiocytic cells, consistent with the fibrohistiocytic nature of dermatofibroma.

Immunohistochemical staining was performed to support the histopathological diagnosis and rule out malignant mimics. The lesional spindle cells exhibited strong cytoplasmic positivity for CD10 (Figure 3A) and CD68 (Figure 3C), confirming the fibrohistiocytic nature of the lesion. In addition, the cells showed diffuse positivity for Factor XIIIa, a marker characteristic of dermal dendritic cells. Notably, the lesion was negative for CD34 (Figure 3B), effectively ruling out dermatofibrosarcoma protuberans, which typically demonstrates strong CD34 positivity. This immunoprofile supports the diagnosis of dermatofibroma.

These histopathological and immunohistochemical findings confirmed the diagnosis of dermatofibroma. Bacterial and fungal cultures of the lesion were negative, excluding infectious etiologies. Given the patient’s advanced comorbidities and poor prognosis, no intervention was pursued for the dermatofibroma, prioritizing the management of her primary illness. The patient later succumbed to complications from pancreatic cancer.

## 3. Discussion

Dermatofibroma typically presents as a firm, asymptomatic nodule, but ulceration and sterile suppuration, as in this case, are rare, occurring in only 6% of cases per Kelati et al. [18]. This presentation in an immunocompromised patient with pancreatic cancer and chemotherapy adds diagnostic complexity. Similar cases in immunosuppressed patients are scarce. Beatrous et al. reported multiple dermatofibromas in patients with autoimmune diseases or HIV, often managed conservatively or with excision [11]. Lungu et al. described an ulcerated dermatofibroma in a 31-year-old male, initially mistaken for keratoacanthoma, treated with excision [11]. In contrast, our patient’s lesion was not excised due to her critical condition, as invasive procedures posed significant risks given her comorbidities and ongoing chemotherapy. Dermatofibromas are benign lesions and only rarely exhibit malignant behavior [20]. However, atypical presentations such as ulceration can clinically mimic malignant melanoma, squamous cell carcinoma, or soft tissue sarcomas [17]. Immunohistochemistry is critical for distinguishing dermatofibromas (Factor XIIIa-positive and CD34-negative) from dermatofibrosarcoma protuberans (CD34-positive and Factor XIIIa-negative) [21]. Excisional biopsy is the standard for diagnosis, with recurrence rare when margins are clear [22]. Additionally, the lesion dimensions and edge characteristics found on histopathology usually correspond closely with sonographic observations. Histologically, a combination of fibroblasts, histiocytes, and thick collagen fibers give dermatofibromas their characteristic appearance [3].

The 4 mm punch biopsy was selected to minimize procedural risks in this frail patient while providing sufficient tissue for histopathological and immunohistochemical analysis [19]. Although excisional biopsy is preferred for ulcerated lesions to ensure adequate sampling, especially when malignancy is suspected, studies suggest punch biopsies can be diagnostic for dermatofibromas when targeted appropriately [17]. In this case, the biopsy confirmed the diagnosis, supported by characteristic collagen trapping and immunohistochemistry.

The ulceration in this case likely resulted from chronic mechanical pressure on the medial thigh, which precipitated localized ischemia and tissue damage, ultimately culminating in epidermal breakdown. This primary insult was compounded by the patient’s diabetes mellitus and chemotherapy-induced vascular compromise; both systemic factors are well known to impair microcirculation and hinder wound healing processes [23,24]. Taken together, these local and systemic factors created an environment conducive to ulcer formation. This pathogenesis aligns with published reports of perforating dermatofibroma, a rare variant characterized by transepidermal elimination (epidermal breakdown) [25]. Notably, perforating dermatofibroma is characterized by lesions that breach the epidermis and ulcerate—a pattern akin to that observed in the present case [26]. Furthermore, repeated cultures of the wound exudate yielded no microbial growth, effectively ruling out an infectious etiology for the ulceration [27]. Consequently, the purulent discharge is best interpreted as sterile suppuration secondary to a robust reactive inflammatory response rather than an active infection [28].

Differential diagnoses included squamous cell carcinoma, basal cell carcinoma, amelanotic melanoma, and cutaneous metastasis. Histopathology was critical, showing epidermal hyperplasia, spindle cell proliferation, and collagen trapping [4]. Immunohistochemistry (CD68-, CD10-, and Factor XIIIa-positive; CD34-negative) distinguished dermatofibroma from dermatofibrosarcoma protuberans, which shows deeper infiltration and CD34 positivity [15,29,30]. Other differentials, like pyoderma gangrenosum or blastomycosis, were ruled out by the absence of neutrophilic infiltration or budding yeast [31]. Although benign, dermatofibromas may clinically resemble malignant melanomas or soft tissue sarcomas—especially in cases with atypical features such as ulceration or erosion [32]. Ulcerated dermatofibroma is a rare variant with damage to the epidermis and superficial dermis, often mistaken for more aggressive lesions. Diagnosis is primarily histopathological, with excisional biopsy being the standard approach; recurrence is rare when excised with intact margins [3].

Management typically involves excision for symptomatic or ambiguous lesions, with low recurrence rates [29]. Alternative treatments, like pulsed dye lasers, have shown 73% patient satisfaction in cosmetically sensitive areas [33]. In this case, the decision to forgo intervention was based on the patient’s poor prognosis and high surgical risk, prioritizing palliative care [34].

Although ultrasound and dermoscopy were not performed in this case, these tools may offer additional diagnostic value in evaluating atypical dermatofibromas. Dermoscopy often shows a central white area with a pigmented rim, while ultrasound reveals avascular lesions with spiculated borders [25,30]. Electrical impedance spectroscopy, explored by Aberg et al., showed limited diagnostic specificity for dermatofibromas [35], whereas fluorescence in situ hybridization can confirm dermatofibrosarcoma protuberans in uncertain cases [36]. These tools were unavailable in this case but could aid future diagnoses [37].

## 4. Conclusions

This case highlights a rare ulcerated and suppurative dermatofibroma in an immunocompromised patient, mimicking malignant or infectious conditions. Accurate diagnosis required integrating clinical, histopathological, and immunohistochemical findings. The decision to avoid intervention was justified by the patient’s critical condition. This report emphasizes considering atypical dermatofibroma variants in ulcerated lesions, particularly in vulnerable populations, and underscores the value of diagnostic tools like dermoscopy and ultrasound when available.

## Data Availability

All data generated or analyzed during this study are presented in this article. For further information, inquiries can be directed to the corresponding author.
